# Keeping Up with Insect Pollinators in Paris

**DOI:** 10.3390/ani12070923

**Published:** 2022-04-04

**Authors:** Vincent Zaninotto, Isabelle Dajoz

**Affiliations:** 1 Institute of Ecology and Environmental Sciences-Paris (iEES-Paris), Sorbonne Université, CNRS, IRD, INRAE, Université Paris Cité, UPEC, 4 Place Jussieu, 75005 Paris, France; isabelle.dajoz@univ-paris-diderot.fr; 2 Direction des Espaces Verts et de l’Environnement, Mairie de Paris, 103 Avenue de France, 75013 Paris, France

**Keywords:** urban pollinators, urban ecology, wild bees, hoverflies, pollinator functional diversity, greenspace management practices, urban biodiversity

## Abstract

**Simple Summary:**

The city of Paris is a dense and highly urbanized capital. However, it has many green spaces, some of which are managed to promote biodiversity. Among the wildlife that can be found in the city, insect pollinators are of great interest because of their pollination services. But what diversity of pollinators can we expect in such an artificial environment? In order to learn more about the species present, we carried out standardized inventories in Parisian green spaces for two consecutive years, over the course of the seasons. We identified 118 species of wild bees and 37 species of hoverflies, some of which had never been observed in Paris before. In particular, we observed relatively high proportions of parasitic and specialist bee species, which are generally uncommon in cities. The greatest diversity was observed in ecologically managed green spaces, suggesting that such approaches effectively support insect communities. Paris is home to many species of pollinators, forming assemblages that shift throughout the seasons. This is evidence that dense metropolises should not be overlooked when it comes to protecting biodiversity.

**Abstract:**

There is growing interest in urban pollinator communities, although they may be subject to biotic homogenization in densely artificial landscapes. Paris (France) is one of the densest cities in the world, yet over the years many insect pollinator species have been reported there. We conducted in-depth surveys of Parisian green spaces for two years, in order to improve our knowledge of these assemblages. We explored several types of green spaces, monitoring pollinators throughout their activity season. We listed 118 species of wild bees and 37 species of hoverflies, updating pre-existing lists with 32 additional species. Bee assemblages showed functional diversity with 18.5% parasitic species and 17.7% oligolectic species. We also found several bee and hoverfly species under special conservation status. Over the study period, we observed seasonal succession of species, with diversified phenological niches. The greatest taxonomic and functional diversity was found in green spaces combining several habitats with ecological management. Despite its very dense urbanism, Paris is home to diverse pollinator communities. As a result, nearly half of the wild bee species of the wider Ile-de-France administrative region can be found within the city. This highlights the need to also consider dense urban environments in insect pollinator conservation strategies.

## 1. Introduction

Urban pollinator communities are receiving increasing attention, and many studies have revealed the potential of urban environments to host a diversity of insect pollinators [1]. More specifically, low-density urban environments are often described as pollinator-friendly [2], especially when they encompass private gardens and allotments [1,3]. As a result, pollinator abundance and diversity can be equally high in urban and rural areas across European landscapes [4,5]. However, urban pollinators are mainly relegated to green spaces, not all of which provide suitable habitat [3]. In green spaces, pollinator communities are under the influence of both local and landscape environmental factors [6]. As demonstrated in Austria [7] and Germany [8], local factors seem to be most relevant, foremost among them flower diversity and abundance. Sufficient floral resources are essential to maintaining functionally diverse bee communities, but are impaired by land artificialization. As a result, in densely built cities, pollinators must cope with a fragmented habitat and scattered resources [9].

Accordingly, in the literature, dense urban areas are often considered as an endpoint of landscape artificialization gradients, and supposedly not suitable for pollinator diversity. Such is the case of several studies measuring the effects of urbanization on bees across France. Although urbanized areas were found to support relatively diverse bee communities [10,11,12], these communities are poorer than those of surrounding suburban and semi-natural areas [2,13,14]. Besides, within dense urban habitats, pollinator assemblages may be subject to community homogenization. As seen in several cities of Poland, the overall urban bee community may only consist of a subset of the rural one, and be identical across all urban landscapes [5]. Such biotic homogenization has also been recorded in France based on a nationwide dataset from participatory science [15]. These results show that the functional composition of pollinator communities is affected by the urban environment, with a shift toward more generalist insect species. Indeed, urban settings are known to apply an environmental filter on pollinators, bees in particular. In the city, species with certain traits seem to be at a disadvantage: such is the case for specialist species, ground-nesting species, large-sized species and early-emerging species [6,16]. These filtering effects of the urban environment on specialist and ground-nesting bee species have also been observed in the Paris region [17,18].

Given its very dense urban landscape, one may expect the city of Paris (France) to be a hostile environment for pollinators. However, over the years of monitoring in the French capital, the assemblage of bee species has proven to be relatively rich. Indeed, from 2011 to 2018, bee monitoring in Parisian green spaces combining two sampling methods (pan traps and nets) has resulted in a list of 119 wild bee species (91 in peer-reviewed studies [18,19,20,21]; and an additional 28 species in ecological assessment reports [22,23]). In contrast, other pollinator taxa have received less attention. Such is the case for hoverflies, with only 47 recorded species in recent studies (17 in published papers [18,24]; and 30 additional species in ecological assessment reports [22]).

Here we present the results of a new inventory of pollinator species in the green spaces of Paris, France. By setting up a standardized monitoring of insect pollinators in 12 green spaces from spring to fall during two consecutive years (2019 and 2020), we aim to: (1) Update the list of bee and hoverfly species in Paris, describing their diversity in several types of green spaces within a high density urban environment; (2) Provide a description of the floral preferences of these species and their conservation status; (3) Assess the various seasonal activity patterns of pollinators from March to October in Paris, to demonstrate the need for extended inventories over multiple seasons.

## 2. Materials and Methods

### 2.1. Features of the City of Paris and the Selected Green Spaces

**Figure 1:**
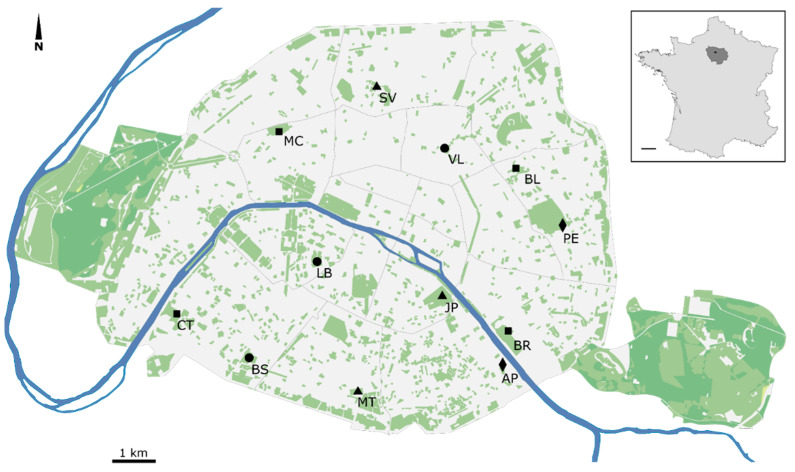
Distribution of the 12 green spaces surveyed in 2019–2020 in the city of Paris. Green: green spaces; grey: built environment; blue: Seine river. Triangles: biodiversity reservoirs with restricted access; diamonds: small ecological parks; squares: biodiversity refuges within large parks; circles: classical gardens. Insert: light grey represents continental France, dark grey represents the Ile-de-France region, which comprises Paris (black dot); the scale bar represents 100 km. Background map: E. Gaba (data: IAU IdF, ODbL, CC BY-SA 4.0, https://commons.wikimedia.org/w/index.php?curid=38508343 (accessed on 15 February 2022)).

This inventory of pollinator species was conducted in the city of Paris, one of the most densely populated cities in the world [25]. Paris covers 105.4 km² and is part of the Ile-de-France administrative region (12,012 km²). Insect collection was carried out in 12 green spaces of different sizes (vegetated areas ranging from 2960 to 116,102 m^2^) evenly distributed throughout the city (Figure 1). These 12 green spaces were integrated into a dense urban matrix, characterized by a high proportion of impervious surfaces (70 to 90% within a 1-km radius) [26]. They had various management practices, but were always free of pesticides. They were classified into four categories: (1) restricted areas, intended to serve as biodiversity reservoirs (MT, SV, JP), managed using ecological practices (no intensive mowing, little pruning, no weeding, only native plant species); (2) small parks open to the public, maintained using the same ecological practices as above, and combining several vegetation layers (AP, PE); (3) large parks with differentiated management including dedicated refuge spaces to accommodate biodiversity (BL, CT, MC, BR); (4) classical gardens with a predominantly ornamental style (preference for horticultural plants and frequent weeding) (BS, LB, VL). The 12 green spaces are managed by diverse stakeholders (for the most part the Paris City Council) and have different histories: some are large historical parks that have undergone transformations over the centuries (MC, JP); other former convents or private mansions (VL, LB, ST); though most are recent parks built on formerly residential or industrial land (AP, BL, BR, BS, CT, PE). MT is located above a buried hydraulic structure dating from 1873. These different histories and management practices appear to result in contrasting herbaceous floral diversity. This and more detailed information on the green spaces surveyed can be found in Appendix A Table A1. 

### 2.2. Standardized Sampling Protocol

We completed a two-years (2019–2020) standardized sampling of insect pollinators with two complementary capture methods. In each green space, we first conducted an active collection of pollinators visiting flowers along a 50 m transect, with two runs at least 10 min apart. All flower visitors were collected within one meter of the transect line using insect nets and plastic boxes. The transect covered several vegetation habitats: grasslands, shrubs, and ornamental flowerbeds; in proportions representative of these respective habitat surfaces in each green space. In addition, we conducted passive insect collection using two sets of colored traps (pan traps-blue/white/yellow cups filled with slightly soapy water) per green space. During each sampling session, these traps were set for two hours.

Sampling sessions were repeated once a month from March to October, in order to get a comprehensive overview of the seasonal successions of pollinator species. April 2020 was skipped due to the COVID-19 crisis. All twelve green spaces were sampled in alternating order during the first two weeks of the month, between 8:00 a.m. and 3:00 p.m. (solar time), under favorable weather conditions (no rain, low wind, temperature > 10 °C).

While distinctly recognizable specimens were identified in the field, most catches were euthanized with ethyl acetate and then carried back to the laboratory. We identified them down to the genus level before sending them to specialists for further identification. The insect specimens are now kept in the collection of the iEES-Paris institute.

### 2.3. Species Traits and Conservation Status

We assessed species conservation statuses from the European red list of bees [27], but also regional lists of bee and hoverfly species that define areas of ecological interest [28,29]. We used several databases to document the traits of the bee species [19,30,31,32,33,34], and hoverfly species [35]. 

## 3. Results

### 3.1. Updated Lists of Bees and Hoverflies in Paris

We found a total of 3142 wild bee individuals, belonging to 118 species. Among these wild bee species, 26 had not been documented in Paris before (Table 1), at least in recent published reports. Therefore, we have completed the list of bee species in Paris from recent reports of 119 wild species [18,19,20,21,22,23], to an updated list of 145 wild species (Supplementary Table S1). We also encountered 1168 domesticated honey bee individuals.

Concerning hoverflies, we collected 394 individuals representing 37 taxa (Table 2). Of these, 6 were new species compared to previous inventories [18,22,24]. We have therefore compiled an updated list of 53 species of hoverflies for the city of Paris (Supplementary Table S2).

Additionally, we found 13 species of Lepidoptera (139 individuals), 4 species of Coleoptera (20 individuals) and three species of bee flies (Bombyliidae, Diptera) (16 individuals). All of them were common species (species list can be found in Appendix A Table A2). We also encountered other Diptera (1101 individuals), other Apocrita (118 individuals), and Symphyta (50 individuals) that we could not identify to the species level.

### 3.2. Specialist and/or Rare Species and Their Habitats

According to the scientific literature, urbanization has a strong negative influence on specialist and parasitic bee species, making their presence notable in a dense city. Here, we encountered bees that are specialized in collecting pollen from plants that belong to a single particular genus: *Andrena florea* (on plants of the *Bryonia* genus); *Andrena ventralis* (*Salix* genus), *Andrena viridescens* (*Veronica* genus), *Hoplitis adunca* (*Echium* genus), *Hylaeus signatus* (*Reseda* genus), *Macropis europaea* (*Lysimachia* genus). We noted the presence of *Colletes hederae*, that collects pollen on only one plant species (monolectic), the ivy *Hedera helix*. We also found some oligolectic bees with broader preferences, but limited to a single plant family: Brassicaceae (the bee *Andrena lagopus*), Fabaceae (*Andrena ovatula*, *Megachile ericetorum*, *Melitta leporina*), Apiaceae (*Andrena proxima*), Campanulaceae (*Chelostoma campanularum* and *Chelostoma rapunculi*), and especially the Asteraceae (*Colletes daviesanus*, *Colletes similis*, *Heriades truncorum*, *Lasioglossum leucozonium*, *Panurgus dentipes*, *Pseudoanthidium nanum*). Furthermore, the hoverfly assemblage also shows a diversity of feeding types, most notably concerning larvae foraging behavior. Thereby we report the presence of herbivorous, predatory, saproxylic and microphagous species (Table 2).

Besides, our sampling includes several genera of brood parasite bees, that deposit their eggs in the nests of the host species, thus appropriating the resources allocated to the larvae (cleptoparasitism). Here, we found brood parasite species covering all major bee nesting types: the bumble bee *Bombus vestalis*, which deposits its eggs in the ground-nesting eusocial *Bombus terrestris* colonies; the bees of the genera *Nomada* (12 species here) and *Sphecodes* (7 species here), which parasitize small non-eusocial ground-nesting bees belonging to the genera *Andrena*, *Halictus* and *Lasioglossum*; and the species *Coelioxys inermis* and *Stelis punctulatissima*, which respectively parasitize the stem-nesting bees of the genus *Megachile* and *Anthidium*. Moreover, the presence of three species of bee flies (Bombylidae, Diptera) should be mentioned. While the adult bee flies feed on nectar and participate in pollination, the maggots are parasitoids of the bee larvae. The eggs are deposited in mid-flight by females directly in the burrows of ground-nesting solitary bees [36]. We also note the presence of commensals such as the hoverfly *Volucella zonaria*, whose detritiphagous and microphagous larvae reside in the nests of eusocial Hymenoptera. Since honey bees and bumblebees seem to thrive in urban areas [37], such commensal species may have the opportunity to prosper in these environments.

Although most of the collected pollinator species are common, some have special conservation status. We recorded 3 bee species under “near-threatened” (NT) status from the European IUCN red list (Table 1). Additionally, 7 bee species (including two of the three NT-European status species) and 4 hoverfly species had special regional statuses in the Ile-de-France, characterizing areas of high ecological interest (Table 2).

Sites that hosted the most specialist bee species were dedicated biodiversity refuge areas within large public parks (BL, CT, MC, BR), followed by the small ecological parks (PE, AP). Results from the natural sites with restricted access were highly variable: the fewest specialist species were found in the SV woodland site; they were more numerous in the MT grassland site; but most were found in JP that combined both habitats (Appendix A Table A1). Species with special conservation status were found at all twelve sites. Nevertheless, most of those conservation status species were found at sites BL and BR, which have high functional diversity of plant assemblages.

### 3.3. Seasonal Distribution of Species

**Figure 2:**
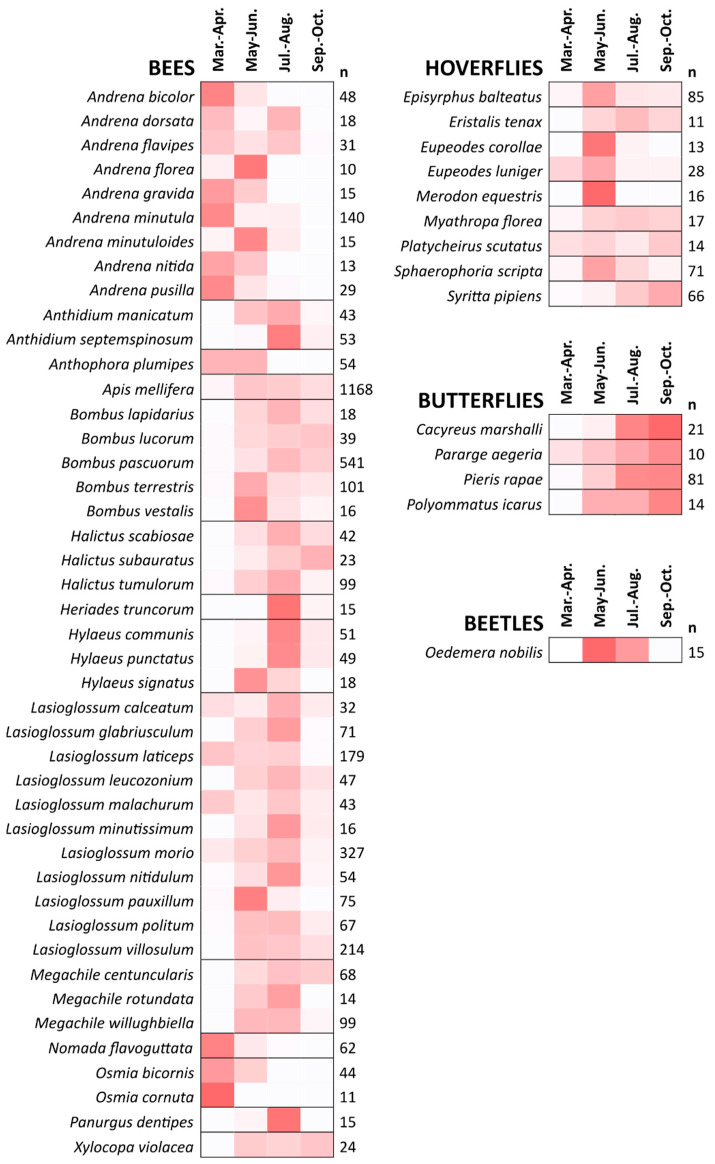
Seasonal distribution of insect occurrence in all twelve green spaces and both years combined. The color intensity gradient represents the distribution of catches for each species over the study period, which is divided into two-month intervals (redder means more catches). Only bee, hoverfly, butterfly, and beetle species collected in large numbers are represented (total number of individuals per species *n* ≥ 10).

Though most bee individuals were collected during late-spring and summer, we observed active bees throughout all seasons from March to October. Indeed, we assessed a seasonal succession of species that resulted in a high temporal complementarity (Figure 2). Some bee taxa displayed broad phenologies of activity, with continuous presence over several months. This is especially true for social species whose colony life cycles last several months, with an increasing number of workers [38,39], such as bumble bees (*Bombus* genus), and primitively social Halictid species (most notably *L. calceatum*, *L. laticeps*, *L. malachurum* and *L. morio*). *Apis mellifera*, the managed honey bee, is present all the time, being the only bee species with a colony cycle that covers all year. Other bee species are only active during a single limited period. Some of them emerge during early spring, such as most of the species in the genus *Andrena*, but also in the genera *Anthophora*, *Nomada* and *Osmia*. Others emerge later, during the summer: this is the case of the genera *Anthidium*, *Heriades*, *Hylaeus*, and *Panurgus*. Besides, we collected bivoltine species from different generations within the year, such as *Andrena dorsata* and *Andrena flavipes* (with a first generation in March-April and another in July-August [40]). This pairing of species with either short or long activity periods was also found in hoverflies. In contrast, the butterfly species commonly found here all show rather long and late activity periods (Figure 2).

## 4. Discussion

Our new sampling extended the list of wild bee species (Anthophila) recently occurring in the city of Paris to 145 species, covering the six bee families found in France and representing 43% of the 340 wild bee species lately found in the wider Ile-de-France region [29] (Supplementary Table S1). We also present a more comprehensive view of the diversity of hoverflies (Syrphidae) in the French capital, with a list of 53 species, representing 25% of the 216 species known in the Ile-de-France region [28] (Supplementary Table S2).

Thanks to repeated surveys over the last decade, and despite the small area of Paris and its high density of urbanization, such pollinator richness is noteworthy compared to other cities. In France, reports often list about a hundred bee species within a city (Marseille: 114 species [12]; Angers: 91 species, Nantes: 134 species, La Roche-sur-Yon: 120 species [11]). In the Lille urban area, Fisogni et al. [13] recorded 102 bee species, as well as 52 hoverfly species. Here, the number of bee species is slightly lower than in other large European cities such as Poznan (206 species) [41] and Zurich (164 bee species) [42], but higher than in North American metropolises (Chicago: 83 bee species [43]; New York City: 98 species [44]), although some variation is likely to arise from climate differences. Furthermore, bee species lists grow considerably larger when surveys are conducted over broader areas encompassing low density suburbs (Lyon region, France: 291 species [2]; Bydgoszcz region, Poland: 242 species [45]).

The Parisian assemblage of wild bee species shows a functional complementarity in trophic resources. Indeed, it includes many generalist species, but also species with specific preferences for various plant families. These communities also suggest complex interactions between pollinator species. Hence, we noted the presence of various brood parasite species, taking advantage of ground-nesting and stem-nesting bees. Parasitic interactions were also recorded between insect orders, as several species of Diptera are able to parasitize bee species. We recorded 22 parasitic bee species, accounting for 18.5% of the total bee species. Yet, parasitic species remained rare as they numbered only 125 individuals (4.0% of the total wild bee abundance). Previous studies in the city of Paris reported a lower percentage of parasitic species (4.5%), suggesting a functionally depleted bee community [21]. Our results, obtained over a wider range of green spaces, rather demonstrate a high occurrence of parasitic species in the French capital, little below the overall rate in France (21%) [2]. This now strong representation of parasitic bees in Paris could be a sign of success in recent management practices favoring biodiversity. Here, the percentage of parasitic bee species is higher than in reports from Lyon (17%) [2], Poznan (12%) [46], and across New York City (15%) [44].

By monitoring the pollinator assemblages over the seasons, we were able to observe a phenological complementarity among species. Some had long flight periods, while others had short flight periods spread throughout the year. The literature suggests that late-emerging species and species with long flight periods may have an advantage in urban settings [1], perhaps because exotic garden plants are mainly providing late-season flower resources [47]. Indeed, patterns of activity at the community level differ between the city of Paris and the surrounding semi-natural rural areas, with a broader period of activity in the city [48]. It is therefore important to consider the phenology of insect pollinators in order to better assess their functional diversity, but also to examine the effects of urbanization on communities. This also underlines the value of conducting pollinator surveys with standardized protocols covering the entire pollination season.

Our results also suggest a positive effect of the ecological management of some Parisian green spaces on pollinator functional diversity. Specifically, small biodiversity refuges within large parks seem to be particularly effective in supporting specialized and rare bee species. Leaving wild unmanaged areas with native flora is one of the key management solutions for promoting pollinator conservation in public green spaces; alongside with reducing mowing and increasing the quantity and diversity of floral resources [49].

## 5. Conclusions

Conservation policies for insect pollinators are developing in France and in the Ile-de-France region where the city of Paris is located [50]. Given the surprising diversity of pollinator communities in a dense urban landscape like Paris, these conservation policies could be strengthened by taking urban spaces into consideration. In Paris, 15 years after the total ban on the use of pesticides in public spaces, managers are committed to increasing vegetation cover and maintaining ecological management areas in order to support biodiversity. Such management practices have proven to enhance the diversity of insect pollinators, as well as plants and birds [51]. They entail favoring the development of wild plant species, among which so-called “weeds”, which can be highly attractive to wild pollinators [3]. Even in a dense urban matrix such as Paris, green spaces can accommodate diverse pollinator communities with remarkable functional diversity. Providing biodiversity refuges within public parks seems to be an effective solution to support these communities.

## Figures and Tables

**Table 1:** Report on the 2019–2020 sampling of bee species in the 12 Parisian green spaces, with numbers of captures for each species. ‘Resources’ describes the method of acquiring pollen (“Poly.”: Polylectic; “Oligo.”: Oligolectic; “Clepto.”: Cleptoparasitic). Species in bold are those which are recorded for the first time in Paris. Superscript numbers indicate possible conservation status: ^1^ Regional ZNIEFF characterizing species; ^2^ IUCN NT conservation status.

Species	Resources	AP	BL	BR	BS	CT	JP	LB	MC	MT	PE	SV	VL
**ANDRENIDAE**													
*Andrena bicolor* (Fabricius 1775)	Poly.	2		12	13	5	7		4	1		4	
*Andrena carantonica* (Pérez 1902)	Poly.		1	1		1							
*Andrena chrysosceles* (Kirby 1802)	Poly.	1									1		
*Andrena cineraria* (Linnaeus 1758)	Poly.			1						1			
*Andrena dorsata* (Kirby 1802)	Poly.	2	1	3	2	1	1	1	3	3			1
*Andrena flavipes* (Panzer 1799)	Poly.		6	3	1	3	4	2	2	10			
***Andrena* *florea*** (Fabricius 1793)	Oligo.						10						
*Andrena gravida* (Imhoff 1832)	Poly.	1	3	1	2		4		1	2	1		
*Andrena haemorrhoa* (Fabricius 1781)	Poly.					1							
*Andrena lagopus* (Latreille 1809)	Oligo.									1			
*Andrena minutula* (Kirby 1802)	Poly.	9	10	39	7	22	24	5	9	5	3	3	4
*Andrena minutuloides* (Perkins 1914)	Poly.		3		4	1	4						3
*Andrena nigroaenea* (Kirby 1802)	Poly.		1	1								1	
*Andrena nitida* (Müller 1776)	Poly.		3		1	2		1	1		1	1	3
***Andrena* *ovatula*** (Kirby 1802) ^2^	Oligo.					1							
*Andrena propinqua* (Schenck 1853)	Poly.					1							
*Andrena proxima* (Kirby 1802)	Oligo.						5						
*Andrena pusilla* (Pérez 1903)	Poly.		7	7	2	10			1				2
***Andrena* *rufula*** (Schmiedeknecht 1883)	Poly.		1	2		1		1					
*Andrena strohmella* (Stoeckhert 1928)	Poly.	1	1				1						
*Andrena subopaca* (Nylander 1848)	Poly.			2									
*Andrena tenuistriata* (Pérez 1895)	Poly.		1				1						
***Andrena* *ventralis*** (Imhoff 1832) ^1^	Oligo.	1											
*Andrena viridescens* (Viereck 1916)	Oligo.		1				1						
*Panurgus dentipes* (Latreille 1811)	Oligo.					2	3			6	4		
**APIDAE**													
*Anthophora plumipes* (Pallas 1772)	Poly.		22	5	8	2		2	4	3	4		4
*Anthophora quadrimaculata* (Panzer 1789)	Poly.					1	3	3	1				
*Apis mellifera* (Linnaeus 1758)	Poly.	47	126	154	158	47	159	143	172	24	43	19	76
*Bombus hortorum* (Linnaeus 1761)	Poly.			1		1							
*Bombus hypnorum* (Linnaeus 1758)	Poly.			1	1				1				
*Bombus lapidarius* (Linnaeus 1758)	Poly.		3	1	4		2	1	3	1	3		
*Bombus lucorum* (Linnaeus 1760)	Poly.		6	9	14	2	1		5		1		1
*Bombus pascuorum* (Scopoli 1763)	Poly.	19	123	41	41	25	27	48	66	3	27	26	95
*Bombus pratorum* (Linnaeus 1760)	Poly.				1		1	1	1		1	2	2
*Bombus terrestris* (Linnaeus 1758)	Poly.		17	16	20	3	5	2	15	5	10	2	6
*Bombus vestalis* (Geoffroy in Fourcroy 1785)	Clepto.		1	1	2				9			3	
*Nomada bifasciata* (Olivier 1811)	Clepto.	1	1			1							
***Nomada* *discrepans*** (Schmiedeknecht 1882) ^1^	Clepto.		1										
***Nomada* *distinguenda*** (Morawitz 1874)	Clepto.		1					1					
*Nomada fabriciana* (Linnaeus 1767)	Clepto.	1		2	1			1		1	1	1	
***Nomada* *ferruginata*** (Linnaeus 1767)	Clepto.			1									
*Nomada flava* (Panzer 1798)	Clepto.			1							1		
*Nomada flavoguttata* (Kirby 1802)	Clepto.	2	4	45		2			3	4			2
***Nomada* *fulvicornis*** (Fabricius 1793)	Clepto.					1	1						
*Nomada goodeniana* (Kirby 1802)	Clepto.												1
*Nomada lathburiana* (Kirby 1802)	Clepto.		1				1						
*Nomada succincta* (Panzer 1798)	Clepto.	1									1		
*Nomada zonata* (Panzer 1798)	Clepto.	1	1	2		1							
*Xylocopa violacea* (Linnaeus 1758)	Poly.		1		10	4	8						1
**COLLETIDAE**													
***Colletes cunicularius*** (Linnaeus 1761)	Poly.									1			
*Colletes daviesanus* (Smith 1846)	Oligo.							1		1			
*Colletes hederae* (Schmidt & Westrich 1993)	Oligo.										1		
***Colletes marginatus*** (Smith 1846)	Poly.									1			
*Colletes similis* (Schenck 1853)	Oligo.				1		2						
***Hylaeus* *brevicornis*** (Nylander 1852)	Poly.						1						
*Hylaeus communis* (Nylander 1852)	Poly.	2	11	2	12	3	2	4	4		2		9
*Hylaeus gredleri* (Förster 1871)	NA						1				1		
*Hylaeus hyalinatus* (Smith 1842)	Poly.	1					1		2	1			
***Hylaeus* *incongruus*** (Förster 1871) ^1^	Poly.					3							
*Hylaeus pictipes* (Nylander 1852)	Poly.		1				1				1		
*Hylaeus punctatus* (Brullé 1832)	Poly.	2	12	9	1		2	1	11	7	3	1	
*Hylaeus signatus* (Panzer 1798)	Oligo.						18						
***Hylaeus* *variegatus*** (Fabricius 1798)	Poly.						1			1			
**HALICTIDAE**													
*Halictus langobardicus* (Blüthgen 1944)	NA					2			1				
***Halictus* *maculatus*** (Smith 1848)	Poly.								1				
*Halictus scabiosae* (Rossi 1790)	Poly.	2	2		7	10	4		1	14			2
*Halictus simplex* (Blüthgen 1923)	Poly.			1	1			1	2		4		
*Halictus subauratus* (Rossi 1792)	Poly.	3	4	1		3	3	4	3		1		1
*Halictus tumulorum* (Linnaeus 1758)	Poly.	13	8	7		6	12	5	43	2	2		1
*Lasioglossum bluethgeni* (Ebmer 1971) ^1^	Poly.			2									
*Lasioglossum calceatum* (Scopoli 1763)	Poly.	1	3	5	5		2	6	1	6	2		1
***Lasioglossum* *cupromicans*** (Pérez 1903)	Poly.						1						
***Lasioglossum* *fulvicorne*** (Kirby 1802)	Poly.							1		1			
*Lasioglossum glabriusculum* (Morawitz 1872)	Poly.			2		1	7		2	59			
***Lasioglossum* *griseolum*** (Morawitz 1872)	Poly.		1										
*Lasioglossum laticeps* (Schenk 1868)	Poly.	9	13	7	22	7	30	10	7	58	11	4	1
*Lasioglossum leucozonium* (Schrank 1781)	Oligo.	7	6			11	2		17	3	1		
*Lasioglossum limbellum* (Morawitz 1876) ^1^	Poly.		1	1			3	1					
*Lasioglossum malachurum* (Kirby 1802)	Poly.	10	6	6	1	6	2	2	1	7	1		1
*Lasioglossum marginatum* (Brullé 1832)	Poly.						2						
*Lasioglossum minutissimum* (Kirby 1802)	Poly.	4	1			3	1	2	1				4
*Lasioglossum morio* (Fabricius 1793)	Poly.	62	39	34	18	8	59	21	24	26	15	20	1
*Lasioglossum nitidulum* (Fabricius 1804)	Poly.	1	5		3		6	19	2	3	11	3	1
*Lasioglossum pallens* (Brullé 1832)	Poly.								1			1	
*Lasioglossum pauxillum* (Schenck 1853)	Poly.	1	41	1	2	1	7		1	16	1		4
*Lasioglossum politum* (Schenck 1853)	Poly.	2	26	1	3	10	11	4	1	3	2	1	3
*Lasioglossum pygmaeum* (Schenck 1853) ^1,2^	Poly.		1	4					1				1
*Lasioglossum sabulosum* (Morawitz 1891) ^1,2^	Poly.			1									
***Lasioglossum* *subhirtum*** (Lepeletier 1841)	Poly.									1			
*Lasioglossum villosulum* (Kirby 1802)	Poly.	13	16	6	4	73	8	1	46	23	9	4	11
***Sphecodes* *crassus*** (Thomson 1870)	Clepto.								1				
***Sphecodes* *ephippius*** (Linnaeus 1767)	Clepto.		1										
*Sphecodes ferruginatus* (Hagens 1882)	Clepto.		1							1	1		
***Sphecodes* *gibbus*** (Linnaeus 1758)	Clepto.									1			
*Sphecodes niger* (Hagens 1874)	Clepto.			1				1					
***Sphecodes* *pseudofasciatus*** (Blüthgen 1925)	Clepto.					1							
***Sphecodes* *puncticeps*** (Thomson 1870)	Clepto.							1					
**MEGACHILIDAE**													
*Anthidiellum strigatum* (Panzer 1805)	Poly.						1		1				
*Anthidium manicatum* (Linnaeus 1758)	Poly.					9	30	2	1		1		
*Anthidium septemspinosum* (Lepeletier 1841)	Poly.		12	6	4		17	1			9		4
*Chelostoma campanularum* (Kirby 1802)	Oligo.						1	1	1				
*Chelostoma rapunculi* (Lepeletier 1841)	Oligo.					3	1						
*Coelioxys inermis* (Kirby 1802)	Clepto.								1		1		1
*Heriades crenulata* (Nylander 1856)	Oligo.		1				1						
*Heriades truncorum* (Linnaeus 1758)	Oligo.		2		1	4		1	5		2		
*Hoplitis adunca* (Panzer 1798)	Oligo.								1				
*Megachile centuncularis* (Linnaeus 1758)	Poly.	4	18	2	7	9	3	2	6		8	4	5
*Megachile ericetorum* (Lepeletier 1841)	Oligo.						2						
*Megachile lagopoda* (Linnaeus 1760)	Poly.		3		3						1		1
***Megachile* *maritima*** (Kirby 1802)	Poly.					1					1		
*Megachile rotundata* (Fabricius 1793)	Poly.	1	1			2	1	1	7		1		
*Megachile willughbiella* (Kirby 1802)	Poly.	7	9	7	11	1	17	8	7	2	20		10
*Osmia bicornis* (Linnaeus 1758)	Poly.	1	7	11	1	3		2	7	3	1	4	4
*Osmia caerulescens* (Linnaeus 1758)	Poly.	1					3		1				
*Osmia cornuta* (Latreille 1805)	Poly.	3	1		1		3			1	2		
***Pseudoanthidium* *nanum*** (Mocsáry 1881)	Oligo.										1		
*Stelis punctulatissima* (Kirby 1802)	Clepto.		3			1			1				
**MELITTIDAE**													
***Macropis* *europaea*** (Warncke 1973)	Oligo.			1									
*Melitta leporina* (Panzer 1799)	Oligo.					3							
**TOTAL (wild bee individuals)**		192	477	316	242	278	383	172	342	288	176	85	191
**TOTAL (wild bee species)**		35	57	47	38	50	59	39	52	39	44	18	33

**Table 2:** Report on the 2019–2020 sampling of hoverfly species (Syphidae, Diptera order) in the 12 Parisian green spaces, with numbers of captures for each species. Larvae feeding type: “H.”: Herbivorous; “P.”: Predator; “M.”: Microphagous; “S.”: Saproxylic. Species in bold are those which are recorded for the first time in Paris. Superscript numbers ^1^ indicate possible conservation status, as a Regional ZNIEFF characterizing species.

Species	Feeding	AP	BL	BR	BS	CT	JP	LB	MC	MT	PE	SV	VL
***Cheilosia grossa*** (Fallén 1817)	H.	1											
*Cheilosia* sp.	H.	1		1		1	1						
*Dasysyrphus albostriatus* (Fallén 1817)	P.										1		
*Epistrophe eligans* (Harris 1780)	P.	1			2								
*Epistrophe nitidicollis* (Meigen 1822)	P.				1						1		
*Episyrphus balteatus* (De Geer 1776)	P.	4	1	8	7	5	4	10	16	1	11	12	6
*Eristalis arbustorum* (Linnaeus 1758)	M.		2				2						
***Eristalis similis*** (Fallén 1817) ^1^	M./S.				1	1	1						1
*Eristalis tenax* (Linnaeus 1758)	M.		5		1		1	2	1		1		
*Eumerus amoenus* (Loew 1848) ^1^	H./M.						2			1	1	1	1
*Eumerus funeralis* (Meigen 1822)	H./M.										1		2
*Eupeodes corollae* (Fabricius 1794)	P.	1	1	1	1				2		2	3	2
*Eupeodes luniger* (Meigen 1822)	P.	2	1	2	4	1		2	4	4	2	2	4
*Eupeodes* sp.	P.				1								
*Helophilus pendulus* (Linnaeus 1758)	M.			4	1		1			1	2		
*Melanostoma mellinum* (Linnaeus 1758)	H./P.				1	1		1					
*Melanostoma scalare* (Fabricius 1794)	H./P.											1	
*Meliscaeva auricollis* (Meigen 1822)	P.			1		2							2
*Merodon equestris* (Fabricius 1794)	H./M.		6		2		2	1	2		3		
*Myathropa florea* (Linnaeus 1758)	M./S.		3	1		2	5		1		3	1	1
*Neoascia podagrica* (Fabricius 1775)	M.				1		1						
***Neocnemodon vitripennis*** (Meigen 1822)	P.						1						
*Paragus haemorrhous* (Meigen 1822) ^1^	P.		1										
*Paragus pecchiolii* (Rondani 1857)	P.											1	
*Pipiza festiva* (Meigen 1822) ^1^	P.	1									1		
*Pipizella* sp.	P.											1	
*Platycheirus scutatus* (Meigen 1822)	P.	1	1	1	3			1	2			3	2
***Platycheirus sticticus*** (Meigen 1822)	P.					1							
*Scaeva pyrastri* (Linnaeus 1758)	P.		1							1			
***Sphaerophoria rueppelli*** (Wiedemann 1830)	P.		1										
*Sphaerophoria scripta* (Linnaeus 1758)	P.	3	8	2	5	6	3	8	10	14	10		2
*Syritta pipiens* (Linnaeus 1758)	M.	16	13	2	1	12	4	9	7				2
***Syrphus rectus*** (Osten-Sacken 1877)	P.							1					
*Syrphus ribesii* (Linnaeus 1758)	P.	1	1		1		1						1
*Syrphus vitripennis* (Meigen 1822)	P.		1		1		1		1		1		
*Volucella zonaria* (Poda 1761)	P./M.								2				
*Xanthogramma dives* (Rondani 1857)	P.					1							
**TOTAL (hoverfly individuals)**		32	46	23	34	33	30	35	48	22	40	25	26
**TOTAL (hoverfly species)**		11	15	10	17	11	15	9	11	6	14	9	12

## Data Availability

All data is contained within the article and Supplementary Materials.

## Supplementary Materials

The following supporting information can be downloaded at: https://www.mdpi.com/article/10.3390/ani12070923/s1, Table S1: Updated list of bee species, summarizing all recently published inventories of Paris (France); Table S2: Updated list of hoverfly species, summarizing all recently published inventories of Paris (France).

## Appendix A

**Table A1:** Additional information on the twelve green spaces surveyed, presented in Figure 1. ‘Habitats’ describes the relative areas of four plant habitats in the green spaces: G, grasslands; S, shrubs; F, flowerbeds; T, tree-covered habitat. Weed species correspond to spontaneous entomophilous herbaceous plant species. We recorded them over the year at each site, in five 1 × 1 m quadrats distributed across the present habitats.

ID	Green Space Name	Coordinates	Vegetated Area (m²)	Habitats (%)				Number of Weed Species
				G	S	F	T	
**Biodiversity reservoirs with restricted access**								
JP	Jardin écologique (MNHN)	48.844°, 2.3614°	11,610	25	20	0	55	30
MT	Réservoir Montsouris	48.8247°, 2.3326°	29,592	100	0	0	0	21
SV	Jardin Saint-Vincent	48.8882°, 2.3413°	2960	0	25	0	75	25
**Small ecological parks**								
AP	Jardin Abbé-Pierre	48.8289°, 2.3803°	11,321	37	52	0	11	22
PE	Jardin Pierre Emmanuel	48.8587°, 2.3989°	5539	8	8	0	84	29
**Biodiversity refuges within large parks**								
BL	Parc de Belleville	48.8714°, 2.385°	37,509	14	18	4	64	22
BR	Parc de Bercy	48.8358°, 2.3827°	116,102	27	14	4	55	23
CT	Parc André Citroen	48.8419°, 2.2746°	77,651	34	22	2	42	30
MC	Parc Monceau	48.8791°, 2.3091°	72,209	32	14	1	53	28
**Classical gardens**								
BS	Parc Georges Brassens	48.8315°, 2.2996°	53,951	24	18	1	57	21
LB	Jardin Catherine Labouré	48.8513°, 2.3208°	4961	57	21	5	17	10
VL	Jardin Villemin	48.875°, 2.3611°	14,702	34	19	6	41	9

**Table A2:** Species lists of flower visitors from the Lepidoptera, Coleoptera and Diptera (Bombyliidae family) orders.

Species	AP	BL	BR	BS	CT	JP	LB	MC	MT	PE	SV	VL
**COLEOPTERA**												
*Cetonia aurata* (Linnaeus 1758)			1	1	1							
*Coccinella septempunctata* (Linnaeus 1758)						1						
*Oedemera nobilis* (Scopoli 1763)			1		3				1	10		
*Trichius fasciatus* (Linnaeus 1758)						1						
**DIPTERA** (Bombyliidae)												
*Bombylius discolor* (Mikan 1796)		1								1		
*Bombylius major* (Linnaeus 1758)		2	1	1		1				3		1
*Villa hottentotta* (Linnaeus 1758)	1		1			2				1		
**LEPIDOPTERA**												
*Aglais urticae* (Linnaeus 1758)				1								
*Cacyreus marshalli* (Butler 1898)	2	4			1		4	1		7		2
*Lampides boeticus* (Linnaeus 1767)							1					
*Lasiommata maera* (Linnaeus 1758)				1								
*Lasiommata megera* (Linnaeus 1767)		2								1		
*Lycaena phlaeas* (Linnaeus 1761)										1		
*Macroglossum stellatarum* (Linnaeus 1758)						1						
*Pararge aegeria* (Linnaeus 1758)	2					4		1		3		
*Pieris rapae* (Linnaeus 1758)	2	10	6	18	3	11	4	7	2	7	6	5
*Polygonia c-album* (Linnaeus 1758)								1				
*Polyommatus icarus* (Rottemburg 1775)		1			1	7			3	2		
*Pyrgus malvae* (Linnaeus 1758)		1										
*Vanessa atalanta* (Linnaeus 1758)			2					1				
